# Temperature, pressure, and duration impacts on the optimal stiffening of carbonates aged in diammonium phosphate solution

**DOI:** 10.1038/s41598-024-57120-z

**Published:** 2024-03-18

**Authors:** Mahmoud Desouky, Murtada Saleh Aljawad, Amao Abduljamiu, Theis Solling, Dhafer Al-Shehri, Murtadha J. AlTammar, Khalid M. Alruwaili

**Affiliations:** 1 Department of Petroleum Engineering, King Fahd University of Petroleum and Minerals, 31261 Dhahran, Saudi Arabia; 2 Center for Integrative Petroleum Research, King Fahd University of Petroleum and Minerals, 31261 Dhahran, Saudi Arabia; 3 EXPEC Advanced Research Center, Saudi Aramco, 31311 Dhahran, Saudi Arabia

**Keywords:** Soft carbonates, Hydroxyapatite, Chalk, DAP treatment, Stiffening, Geochemistry, Mineralogy, Petrology, Surface chemistry

## Abstract

Diammonium phosphate (DAP) has been proven effective in improving the stiffness of weak or acid-damaged carbonates, thereby preserving hydraulic fracture conductivity. The reaction between DAP and calcite in chalk formations primarily produces hydroxyapatite (HAP), which is stiffer than calcite. However, the optimal reaction outcomes vary greatly with factors such as DAP concentration and reaction conditions. This study investigated the DAP-calcite reaction duration, pressure, and temperature effects on the stiffness magnitude of soft Austin chalk. Also, the catalyst effect and depth of HAP formation were examined. The study involved the assessment of stiffness non-destructively (impulse hammering), mineralogy (XRD, SEM), and elemental composition (XRF). The study tested 15 different DAP-chalk reaction variations, where the pressure, temperature, aging time and catalyst addition were modified in each case. The samples' elastic stiffness distributions were then collected and compared to the pre-reaction ones. The results showed that the elastic stiffness increased in all treated samples, with an 181% maximum increase achieved after 72 h at 6.9 MPa and 75 °C. However, the pressure effect was minor compared to the temperature. The SEM images revealed different HAP morphology corresponding to different treatment conditions. Although the treated samples showed an increased intensity of phosphorus throughout the entire sample, the near-surface zone (4–6 mm) was the most affected, as inferred from the XRF elemental analysis. The study's findings can help optimize hydraulic fracturing operations in weak carbonate reservoirs, improving production rates and overall well performance.

## Introduction

Maintaining fracture conductivity is crucial to hydraulic fracturing operations, where rock stiffness greatly impacts^1^. Minerals are the rocks' building blocks; their composition and crystal structure uniquely define them^2^. At the mineral level, hardness is determined by the strength of the chemical bonding and is related to a mineral's resistance to scratching^3^. For example, calcite and apatite have 3 and 5 on Moh's scale, respectively^4^. In comparison, macro hardness is the ability to resist deformation, such as indentation, which may be related to how the grains are cemented^5^. Constituent minerals contribute to the overall hardness of the rock they form^6,7^. Therefore, the minerals forming the rock and how the grains are cemented reflect the final rock's elastic stiffness^8^. As long as calcium (Ca) and phosphate (PO_4_) ions exist, apatite can exist in various forms. Thus, replacing a relatively soft mineral, i.e., calcite, with a harder one, i.e., apatite, can result in stronger structures than the original^9^. Yaşar and Erdoğan^10^ investigated the relationship between hardness value and physicomechanical properties of constructional and cover rocks and concluded that physicomechanical properties can be estimated using hardness methods. In the context of hydraulic fracture conductivity, stiffness is a superior and more precise indicator of a rock's mechanical properties compared to macro hardness. While there may be some correlation between the two in certain types of rocks, stiffness measurements provide a more comprehensive understanding of the rock's mechanical behavior. Stiffness directly correlates with the rock's ability to withstand deformation, making it a more reliable indicator of its capacity to endure applied pressure and retain its shape during hydraulic fracturing.

Several methods are commonly used to prepare HAP (a specific type of apatite mineral that contains hydroxide ions OH^−^), including hydrothermal, precipitation, solvothermal, spontaneous combustion, micro-emulsion, ultrasonic synthesis, bionic and solid-state reaction, or wet and dry methods^11^. On the one hand, the precipitation process used in the formation of HAP from calcite typically involves the reaction of calcium carbonate with a phosphate source, such as diammonium hydrogen phosphate (NH_4_H_2_PO_4_), known as DAP, in the presence of water^12^. On the other hand, hydrothermal is similar to precipitation but at high temperatures and pressures^13^. Also, researchers suggested that the HAP crystals can be synthesized from calcite crystals through hydrothermal treatment. Previous research conducted by multiple authors has demonstrated the successful synthesis of HAP crystals using the method described^14–16^.

Different synthesis approaches result in different HAP crystal structures. The shape variations of HAP are attributed to the distinct reactivity level of each exposed calcite crystal plane towards the surrounding solution^17^. For instance, the (100) plane of calcite is more reactive than the (111) plane, which has a thicker coverage of newly formed HAP. The shape of HAP crystals produced under hydrothermal conditions is affected by several factors, including the solubility of the starting material, processing temperature, pH of the solvent additives, residence period, and others^18^. Also, the concentration of the reactants plays a crucial role in determining the morphology of HAP crystals. Higher concentrations of DAP and CaCO_3_ can promote faster nucleation and growth of HAP crystals, forming smaller crystal sizes and different morphologies^12^.

The hydrothermal process is a commonly used method for the synthesis of HAP from calcite, as it allows for the control of the particle size, morphology, and crystallinity of the HAP product. As a result, the HAP produced by this method is widely used in various applications. For example, hydrothermally-formed HAP has prosthetic and other biomedical applications^19,20^. Also, Pai et al.^21^ reviewed the use of HAP and its composites as adsorbents for removing pollutants from wastewater, including heavy metals, organic compounds, and dyes. They described the mechanisms of adsorption and the factors that affect the adsorption performance, such as pH, temperature, and initial pollutant concentration. In addition, Ashokan et al.^18^ described a dissolution precipitation method to produce HAP microspheres for Chromatographic uses. In art preservation, using the DAP has proved to be a promising technique in restoring the aesthetic and mechanical integrity of damaged carbonate rock^22^. According to recent findings by Murru and Fort^23^, the immersion of limestone in DAP has resulted in a significant enhancement of mechanical performance, which was assessed through variations in ultrasonic measurements.

In recent experiments conducted by Samarkin et al.^24^, hydrothermal treatment was utilized to form HAP in weak chalk samples for use in petroleum applications. The purpose of this technique was to enhance fracture surface elastic stiffness and sustain hydraulic fracture conductivity. The results of the experiment revealed that the surface elastic stiffness of the weak chalk samples was significantly improved, ultimately leading to a higher hydraulic conductivity of the fractures^25–27^. These findings offer valuable insight into the potential for HAP to improve the effectiveness of hydraulic fracturing techniques in the petroleum industry. Such developments hold the promise of enhancing the industry's efficiency and reducing costs.

Thermodynamics favors the mineralogical exchange to convert calcium carbonate into HAP by DAP treatment^18^. However, little is known about the time scale for the exchange at reservoir conditions (i.e., high pressure and high temperature). A fast reaction is favored for the process to be applicable in petroleum-related operations. Therefore, the DAP-calcite reaction and HAP generation speed must be addressed and fully understood. This study aimed to determine the effectiveness of strengthening chalk specimens by adjusting key factors such as pressure, temperature, and time in the formation of HAP. Additionally, the study conducted a detailed analysis of the distribution of HAP in the treated chalk samples. Finally, the research investigated the catalytic effect of Li ions on the DAP reaction with calcite.

## Materials and methods

### Rock samples and chemicals

**Figure 1:**
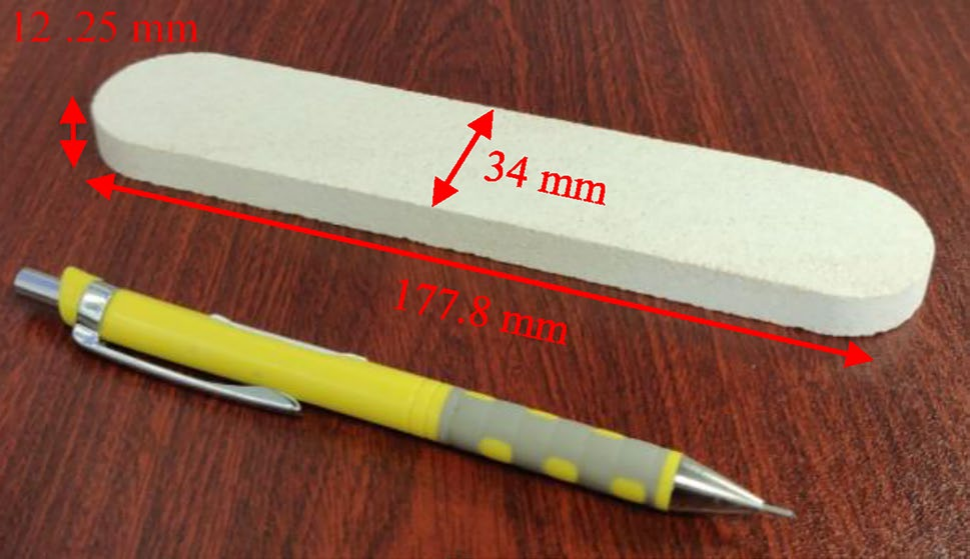
Austin chalk sample.

The experimental work in this study was conducted using 15 slabs of Austin chalk, which was chosen as reference material due to its low stiffness. The Austin chalk samples are composed mainly of calcite, with a purity level of 99.4%. The samples contained negligible other minerals (~ 0.6%), with identification beyond the main constituent falling outside the scope of this study. The slabs have dimensions of 177.8 mm in length, 38.1 mm in width, and 12.7 mm in thickness, as shown in Fig. 1. Two chemicals were used in the current study. The first is a high-purity DAP ((NH_4_)_2_HPO_4_) from Sigma Aldrich, with a purity exceeding 99%. This water-soluble salt serves as a chalk consolidation agent in the present study. The other one is lithium nitrate hydrate (LNH), a chemical compound with the formula LiNO_3_·3H_2_O. It is an odorless, colorless, and water-soluble salt primarily used as a precursor to produce other lithium compounds, including lithium carbonate. In this research, LNH was tested for its potential to catalyze the DAP-chalk reaction. The aim was to accelerate the creation of HAP by catalyzing the exchange process using direct methods and identifying the conditions that promote bonding between DAP and carbonate. The method focused on enhancing the interaction between an electron donor and an acceptor. Therefore, Li^+^ was introduced to improve the electron acceptor (carbonate) by adhering to the O of the C=O group and drawing electrons from the carbon. The potential for lithium ions (Li^+^) to enhance the interaction between diammonium hydrogen phosphate (DAP) and calcium carbonate (chalk) in the stiffening process was investigated. Based on the hypothesis that Li^+^ could facilitate DAP attachment to the carbonate surface, its catalytic effect was incorporated into the study design. To explore this potential, 5 g of lithium nitrate hydrate (LiNO_3_·H_2_O) was added per liter of the 1 M DAP solution, allowing for evaluation of its ability to accelerate the DAP-induced stiffening observed in the chalk slabs.

Calcite's solubility in water is naturally low in the order of hundreds of mg/L^28,29^ and further hampered by relatively high pH, such as in 1 M DAP^30^. Therefore, using 5 g/L of lithium nitrate hydrate (LiNO_3_·3H_2_O) as a catalyst seems excessive. Typically, effective catalysts work in the parts per thousand (per mil ‰) range, requiring minimal amounts where they act as a facilitator, enhancing reactions without being consumed. So, if it has any effect at all, even a few milligrams could effectively catalyze the reaction, offering a cost-effective approach.

### Procedure and instruments

#### Pretreatment

This study employed the New England Research AutoScan impulse hammering technique to analyze the elastic properties of the chalk samples. This method utilizes a freely falling mass (Impulse Hammer) that impacts the sample surface, generating a force–time response measured by a sensor. An elastic Hertzian solution applies this data to estimate the reduced elastic stiffness, a parameter reflecting the mechanical behavior of the core. This non-destructive approach allows for spatially resolved profiling of geomechanical properties, offering insights into strength and elasticity across the sample surface^31^. Multiple measurements (30 points) were conducted uniformly across each sample area to capture potential variations (Fig. 1).

Also, the chalk's composition, structure, and morphology were thoroughly analyzed using a combination of X-ray fluorescence (XRF), X-ray diffraction (XRD), and scanning electron microscope (SEM) techniques.

First, determining the depth of a chemical reaction within a solid surface can be a complex process that requires various techniques, depending on the type of reaction and the surface being studied. The XRF is a valuable tool for exploring the distribution and formation of minerals in porous media. Therefore, Bruker M4 Tornado XRF was utilized for the elemental distribution of a sample with dimensions of 34 × 76.2 × 177.8 mm. It is a non-destructive tool widely used in geology and environmental science, providing valuable information about diverse materials' elemental composition and mapping.

Second, mineralogical characterization of the chalk samples was done with powder XRD. A finely ground powder was obtained from a small portion of the untreated chalk; the powder was then analyzed using the PANalytical Empyrean diffractometer, an advanced XRD instrument within a 2θ range of 0°–70°. The SEM, XRF, and XRD analyses provided valuable insights into the chalk's composition, structure, and morphology when contrasted with the results of the untreated samples.

Finally, small cubes were extracted from the untreated samples to be imaged at the nanoscale level with Zeiss Gemini 450 SEM at the Center for Integrative Petroleum Research, King Fahd University of Petroleum and Minerals Dhahran, Saudi Arabia.

#### Treatment

Once the elastic stiffness of the samples had been evaluated, they were subjected to one of the aging conditions specified in Table 1 to study their impacts on the stiffening. The sample's treatment is conducted by placing it in a Hastelloy steel cell for 24 h (hours) following vacuuming of the cell. In the first set of experiments, 9 samples with similar initial elastic stiffness distribution, 3.7 GPa on average, were aged at different pressure and temperature combinations. Every sample was treated at ambient, 6.9, or 13.8 MPa pressure at each temperature inside a 3L-aging cell filled with 1 M DAP. Following the treatment, the samples were extracted and rinsed with deionized water to eliminate any residual treatment solution. The samples were then dried for 24 h in an oven at a temperature of 75 °C. The aging process is highly similar to the hydrothermal synthesis used to synthesize various materials by subjecting reactants to high-pressure and high-temperature conditions in an autoclave closed vessel. The method is widely used in materials science and engineering, particularly for synthesizing inorganic materials such as HAP from DAP-calcite reactions^14–16^. In the subsequent experiments, three samples underwent DAP treatments at an increment in treatment time (6, 18, 36, and 72 h) at 6.9 MPa and 75 °C. After each treatment interval, the samples were taken out of the aging cell, washed thoroughly with DI water, and dried in the oven. The same procedure was repeated for all the planned intervals, where the residual effluents from each previous interval were used in the subsequent one.

**Table 1:** Different treatments design.

Sample ID	Treatment condition				
	Concentration (M)	Temperature (°C)	Pressure (MPa)	Catalyst	Time (h)
S#1	1	24	0	–	72
S#2			6.9		
S#3			13.8		
S#4		75	0		
S#5			6.9		
S#6			13.8		
S#7		120	0		
S#8			6.9		
S#9			13.8		
S#10		75	13.8		6, 18, 36, and 72
S#11					
S#12					
S#13				LiNO_3_·3H_2_O	6
S#14					
S#15					

For the final set of experiments, three samples with comparable elastic stiffness were subjected to an identical treatment using 1 M DAP for 6 h at 6.9 MPa and 75 °C, but with the addition of 5 g of LNH per liter of DAP. The remaining procedures were carried out in the same manner as the previous experiments.

The field conditions closely resemble the parameters investigated in this study, including comparable temperature and pressure levels. Conventional carbonate reservoirs demonstrate significant variability in pressure and temperature due to their diverse geological contexts^32^. Equinor's analysis of a global database of 100,000 petroleum reservoirs revealed that the majority of conventional oil (97%) and conventional gas (90%) are typically found in reservoirs with temperatures below 120 °C. This temperature range, known as the “Golden Zone”, exhibits a high concentration of resources^33^. Most of these reservoir observations exhibit normal pressure conditions, with approximately 79% or four out of five reservoirs within the Golden Zone characterized by both normal temperature and normal pressure^32^.

#### Post-treatment

The post-treatment analysis involved conducting a comprehensive examination of the chalk samples after the aging process. Firstly, the surface elastic stiffness of the samples was reassessed to determine the effects of different aging conditions.

In addition, another sample with dimensions of 34 × 76.2 × 177.8 mm underwent a 72-h treatment in an aging cell at a temperature of 75 °C and a pressure of 6.9 MPa. The purpose of this treatment was to investigate the DAP reaction and the bulk of the sample. The invasion depth of the DAP reaction was assessed by cutting the sample across its length. Subsequently, XRF scans were performed to compare the elemental composition of the treated sample with that of the original intact sample.

Furthermore, depth profiling was carried out to determine the extent of the reaction. The profiling involved measuring the concentration of phosphorus, which is an indicator of HAP formation, across and along the sample's surface at five different lines. By tracking the presence of phosphorus, the concentration of HAP could be determined, shedding light on the reaction depth and distribution.

To gain further insights into the treated chalk samples, a finely ground powder was obtained from a small portion of the treated chalk. The XRD spectrum of this powder was then compared to the benchmark XRD spectrum of the untreated powder. This comparison provided valuable information about the changes in mineralogical composition resulting from the treatment.

**Figure 2:**
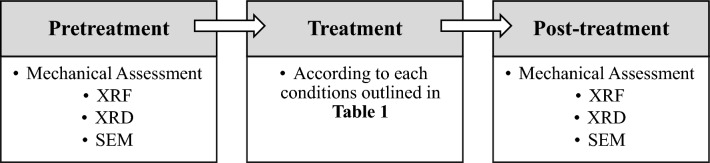
Workflow of the experimental analyses.

Finally, small cubes were extracted from the treated samples for analysis using a scanning electron microscope (SEM). SEM analysis is particularly useful for characterizing and examining the nanoscale structure and behavior of hydrothermally formed HAP. The synthesis of HAP from DAP and CaCO_3_ can lead to various HAP morphologies influenced by reaction conditions, reactant concentrations, and the presence of additives^12,34^. Therefore, by studying the treated samples with SEM, valuable insights into the nanoscale structure and behavior of the hydrothermally formed HAP could be obtained. The comprehensive experimental procedure is depicted in Fig. 2.

### Statistical significance

The elastic stiffness of chalk varies randomly along the surface within a certain range, making it mandatory to statistically validate the elastic stiffness improvement. The repeated experiments were intentionally done to reduce the uncertainty in the obtained results and to acquire more data to validate the results statistically. Therefore, statistical significance testing determines whether the elastic stiffness improvement was due to the treatment or just the result of a chance. Thus, the analysis of variance (ANOVA) technique was used to compare the means of treated and untreated elastic stiffness distributions to reveal if the treatments significantly affected chalk elastic stiffness. In addition, Tukey's HSD was utilized when statistical significance was present. Tukey's HSD is a statistical method used to compare the means of multiple groups and determine which groups are significantly different from each other. Furthermore, pair-wise comparisons were generated to visually represent this test's results, displaying the mean difference between each group alongside confidence intervals and significance levels. Where the confidence interval excludes zero, statistical significance exists between the two groups at the chosen significance level (generally 0.05). The data visualization and computations were made in Rstudio 2023.03.1.

## Results

### Temperature–pressure interaction effect on stiffness

**Figure 3:**
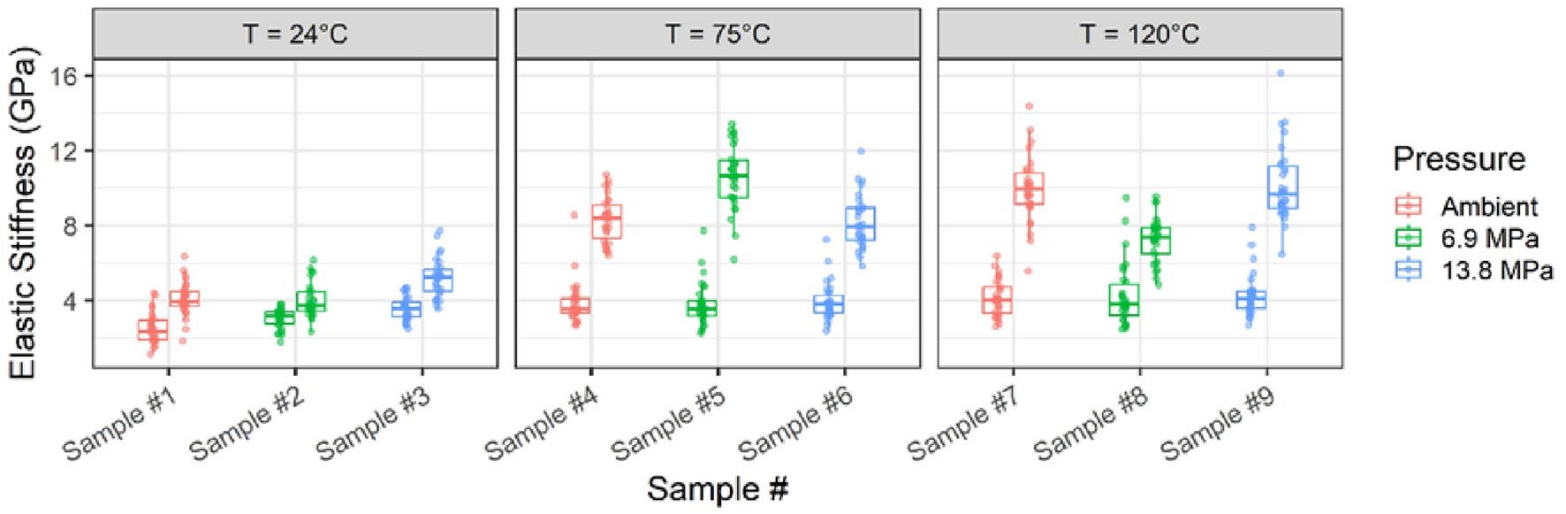
Pre- and post-elastic stiffness distribution under varying pressure and temperature treatments.

The various treatments increased the 'samples' elastic stiffness, as illustrated in Fig. 3. Each sample has two distributions: pre-elastic stiffness on the left and post-elastic stiffness on the right. However, the extent of this increase varied depending on the temperature and pressure conditions. Specifically, the effect of temperature on elastic stiffness was evident when comparing samples treated at elevated temperatures (75 and 120 °C) to those treated at lab ambient temperature (24 °C). At 13.8 MPa and 24, 75, and 120 °C temperatures, average elastic stiffness increased by 46%, 108%, and 138%, respectively. A similar trend was observed across different temperatures and ambient pressures. However, the stiffness of Sample #5 exceeded expectations, exhibiting a larger-than-anticipated increase of 181% following treatment at 75 °C and 6.9 MPa. Conversely, the increase in elastic stiffness of Sample #8, treated at 75 °C and 13.8 MPa, appeared to be less than expected. In contrast, the increased elastic stiffness with the increase in treatment pressure did not exhibit a clear trend at different temperatures.

A one-way ANOVA analysis was performed to investigate the individual effects of different factors on elastic stiffness, including the sample, pressure, temperature, and pre/post-elastic stiffness. Notably, the sample, temperature, and pre/post-elastic stiffness were identified as significant variables impacting the measured elastic stiffness. The results of the one-way ANOVA analysis revealed the crucial role played by these factors in determining stiffness measurements. For instance, the analysis indicated that the pre/post-elastic stiffness factor effectively captured changes in stiffness resulting from the treatment.

In contrast, two-way ANOVA examines the effects of two independent variables on a dependent variable. The one-way ANOVA results demonstrated that the sample, temperature, and pre/post-elastic stiffness all had a significant impact on elastic stiffness. However, the impact of pressure, while statistically significant, was relatively minor compared to the influence of temperature.

Additionally, a two-way ANOVA analysis was conducted to explore the effects of pressure, temperature, and their interaction on elastic stiffness. The results, presented in Table 2, indicated that all three factors had a significant influence on the elastic stiffness.

**Table 2:** Two-way ANOVA analysis of temperature and pressure as factors.

Factor	Degree of freedom	Sum sq.	Mean sq.	F value	Pr(> F)	*p*
Temperature	2	427	213.28	32.535	4.69e−14	< 0.001
Pressure	2	99	49.72	7.585	0.000565	< 0.001
Temperature: pressure	4	284	70.99	10.830	< 2e−16	< 0.001

**Figure 4:**
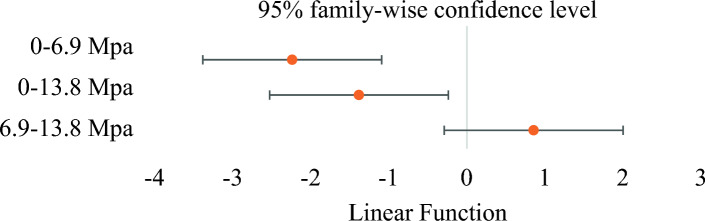
Tukey's HSD for comparison among different pressure groups.

In addition, Tukey's HSD was utilized to analyze the differences in elastic stiffness means associated with pressure. The Tukey family-wise confidence level is typically expressed as a percentage. It represents the probability that the confidence interval captures the true difference between any two groups in a multiple comparison test. Setting the Tukey family-wise confidence level at 95% means that there is a 95% probability that the confidence interval determined for each pairwise comparison contains the actual difference between the means of the groups. Tukey's HSD analysis shows that the mean elastic stiffness for 0 MPa pressure significantly differed from that for pressure levels of 6.9 and 13.8 MPa. However, no significant difference between 6.9 and 13.8 MPa pressure was observed as the confidence level contains 0 (see Fig. 4).

The statistical study showed that the temperature positively affected chalk elastic stiffness after DAP treatment, whereas the pressure had a slightly negative impact. The usual reservoir pressure and temperature values vary greatly depending on the reservoir and location. In addition, the combination of pressure and temperature of 6.9 MPa and 75 °C significantly increased the elastic stiffness of the surface of chalk. Therefore, these precise parameters were used in the next experiments to imitate downhole circumstances better.

### Treatment duration effect on stiffness

**Figure 5:**
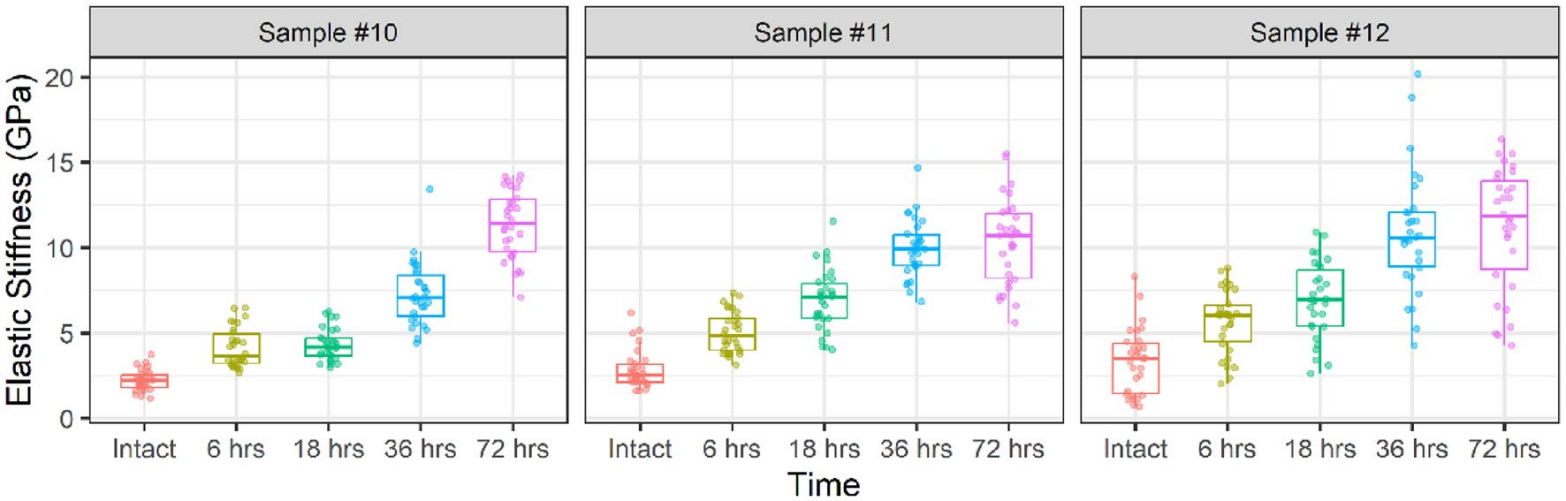
Pre- and post-elastic stiffness distribution under increasing time treatments.

The results showed a clear and consistent increase in elastic stiffness across all three experiments, with the elastic stiffness of the samples increasing as they were treated for longer periods (see Fig. 5). At 72 h, the samples exhibited the greatest increase in elastic stiffness, and there were no indications of stabilization, indicating that there is potential for further increases in elastic stiffness if the treatment were to be extended for a longer period. Specifically, at the 72-h mark, the increase in average elastic stiffness for Sample #10, Sample #11, and Sample #12 was 411%, 261%, and 233%, respectively.

### Catalysis effect on stiffness

**Figure 6:**
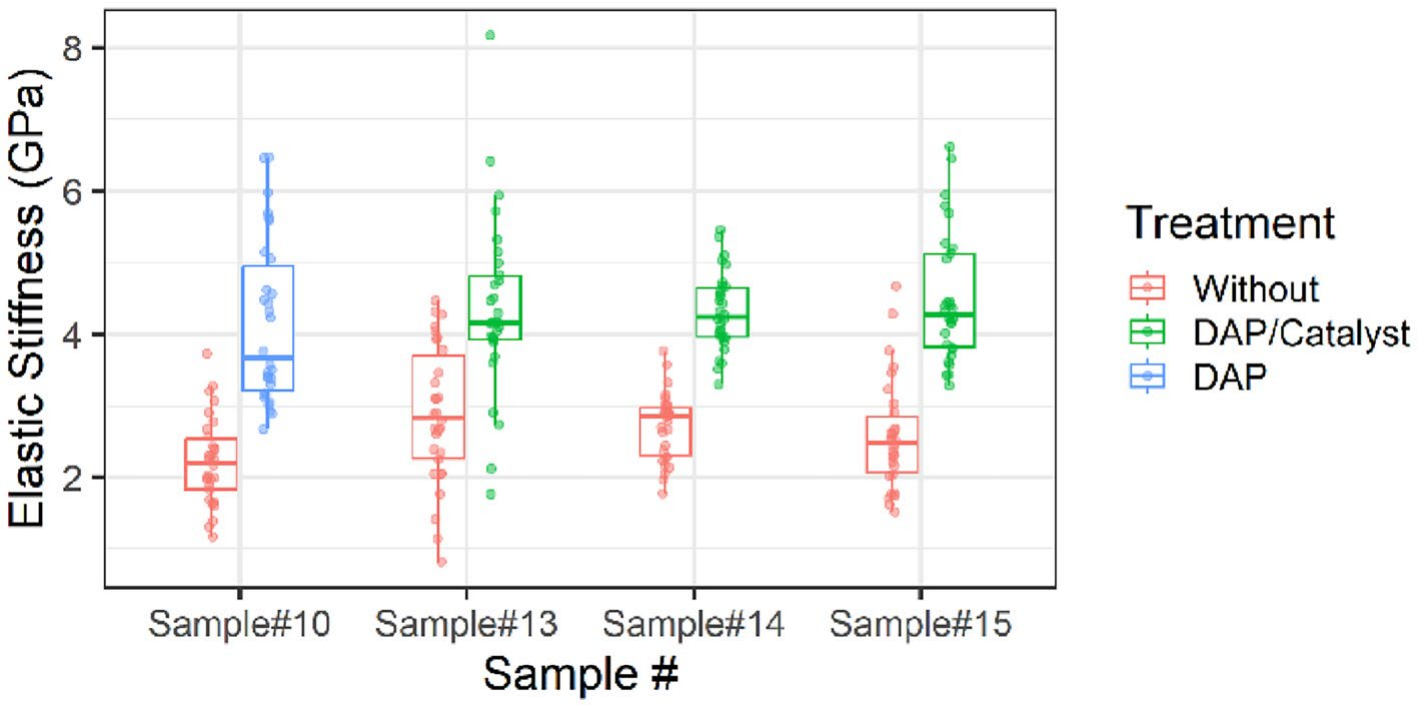
Pre- and Post-elastic stiffness distribution for catalyzed and uncatalyzed treatments.

After undergoing a 6-h treatment, Samples #13, #14, and #15 displayed an increase in average elastic stiffness. However, no tangible difference was visually observed when comparing the elastic stiffness distributions of the three treated samples with the LNH in the DAP solution to that treated with DAP only (Sample #10), as depicted in Fig. 6. The three samples subjected to treatment with LNH catalysis exhibited an increase in elastic stiffness on average of 84% compared to 61% in the treated sample without LNH.

Therefore, ANOVA analysis was conducted to validate the results statistically, where the results are arranged in Table 3. The effect of the treatment is statistically significant and large, but the catalyst effect is significant and small. This finding suggests that the LNH slightly impacted the elastic stiffness properties of the sample. In this case, the result is significant in the sense that it is unlikely to have occurred by chance, but it may be small enough that it is not practically significant.

**Table 3:** Two-way ANOVA analysis of temperature and pressure as factors.

Factor	Degree of freedom	Sum sq.	Mean sq.	F value	Pr(> F)	*p*
Pre/post-elastic stiffness	1	178.9	178.86	238.181	< 2e−16	< 0.001
Catalyst	1	6.2	6.20	8.258	0.00443	< 0.001

### Determination of reaction depth using XRF

**Figure 7:**
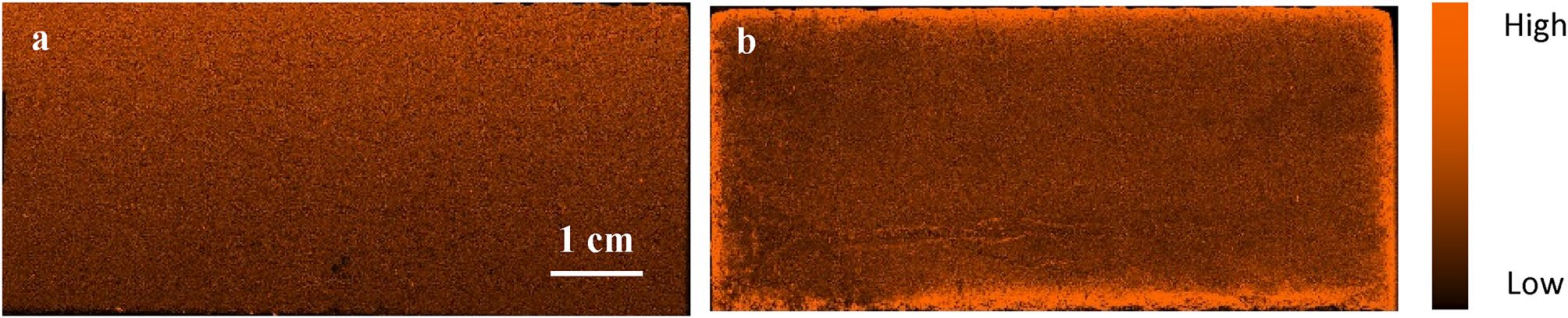
Pseudo-colored images showing phosphorous concentration in (**a**) Untreated, (**b**) treated chalk samples.

In the XRF analysis, a pseudo-orange color was utilized to denote phosphorous, where the orange hue gradient represents the variation in phosphorous concentration. A brighter color indicates a higher phosphorous concentration, which indicates the presence of HAP and vice versa (see Fig. 7a,b). As a result, the treated sample displayed an increased phosphorous intensity across the surface (Fig. 7b). In addition, a higher intensity of the color was observed along the edges of the sample, indicating a higher phosphorous content.

**Figure 8:**
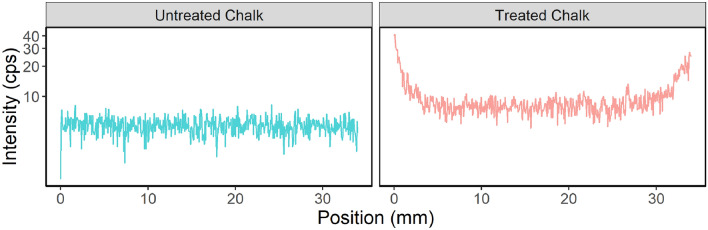
Intensity of phosphorous with location across the untreated and treated samples.

Then, the average intensity values were plotted against the position. The treated sample showed higher phosphorous intensity in the interior, with an average value of 8 cps compared to an average value of 5 cps corresponding to the untreated sample. Also, the treated sample showed a noticeable increase in gradient towards the edges, with the effect becoming more pronounced at a distance of 5 mm from the edge, as shown in Fig. 8. The level of phosphorous intensity reaches its peak at the superficial layer when it comes in direct contact with the solution, with values of up to 40 cps recorded.

### Characterization of mineral composition using XRD and EDS

**Figure 9:**
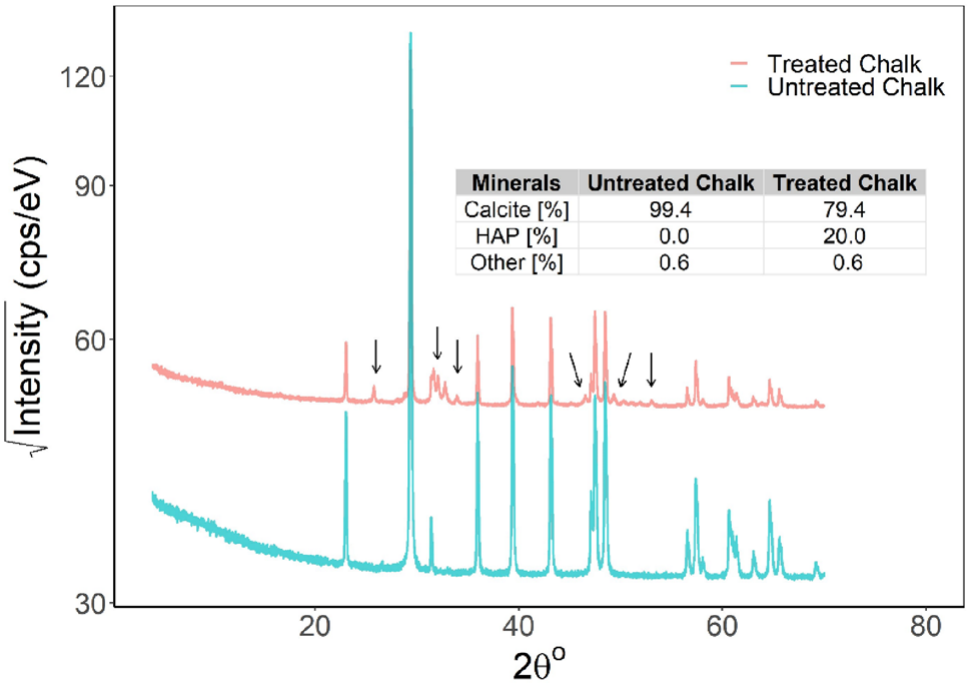
XRD spectra of powder from treated and untreated chalk samples.

Figure 9 presents the XRD spectra of the untreated chalk and the treated chalk powder. Both spectra have been plotted on a single figure to analyze the impact of the DAP treatment on the baseline chalk mineralogy. The spectrum corresponding to the treated chalk displays new peaks at approximately 2θ values of 26, 32, 34, 46, 50, and 53, annotated with black arrows in the plot.

Furthermore, according to the EDS analysis, the virgin sample had a high calcite content, approaching purity with over + 99% calcite present. In comparison, the treated samples showed a significant increase in HAP percentage, representing approximately 20% of the extracted sample near the surface. The sample contained a minimal amount of other minerals, approximately 0.6%. It is important to note that the primary focus of this study was not to identify these minerals individually despite their presence.

### Morphological changes observed with SEM

**Figure 10:**
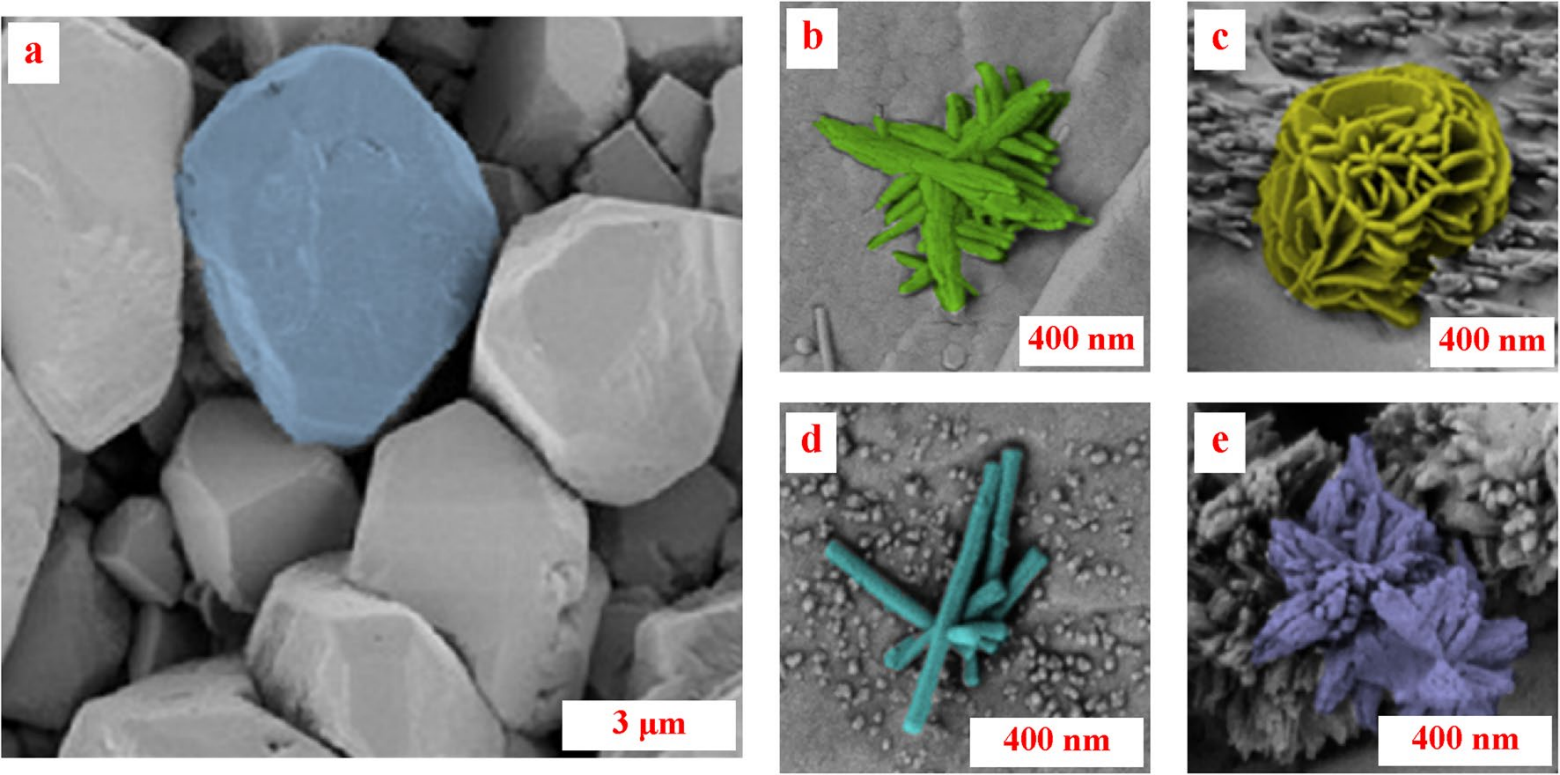
Colorized SEM images of (**a**) intact separate calcite grains, (**b**) Needle-shape HAP crystals, (**c**) Rose-shape HAP crystals, (**d**) Rod-shape HAP crystals, (**e**) Spikey-shape HAP crystals.

Initially, a sample of untreated chalk was scanned to represent the unaltered calcite grain structure, as illustrated in Fig. 10a. The scans showed that individual grains are distinct and exhibit differences in size and shape. Next, the treated samples were scanned to visualize the DAP-induced alterations in the calcite. The SEM images revealed the presence of heterogenous morphologies of HAP in shape and size, as shown in Fig. 10b–e. The formed HAP shapes are a few hundred nanometers with various shapes, including needle-like, rod-like, plate-like, and rose-like.

**Figure 11:**
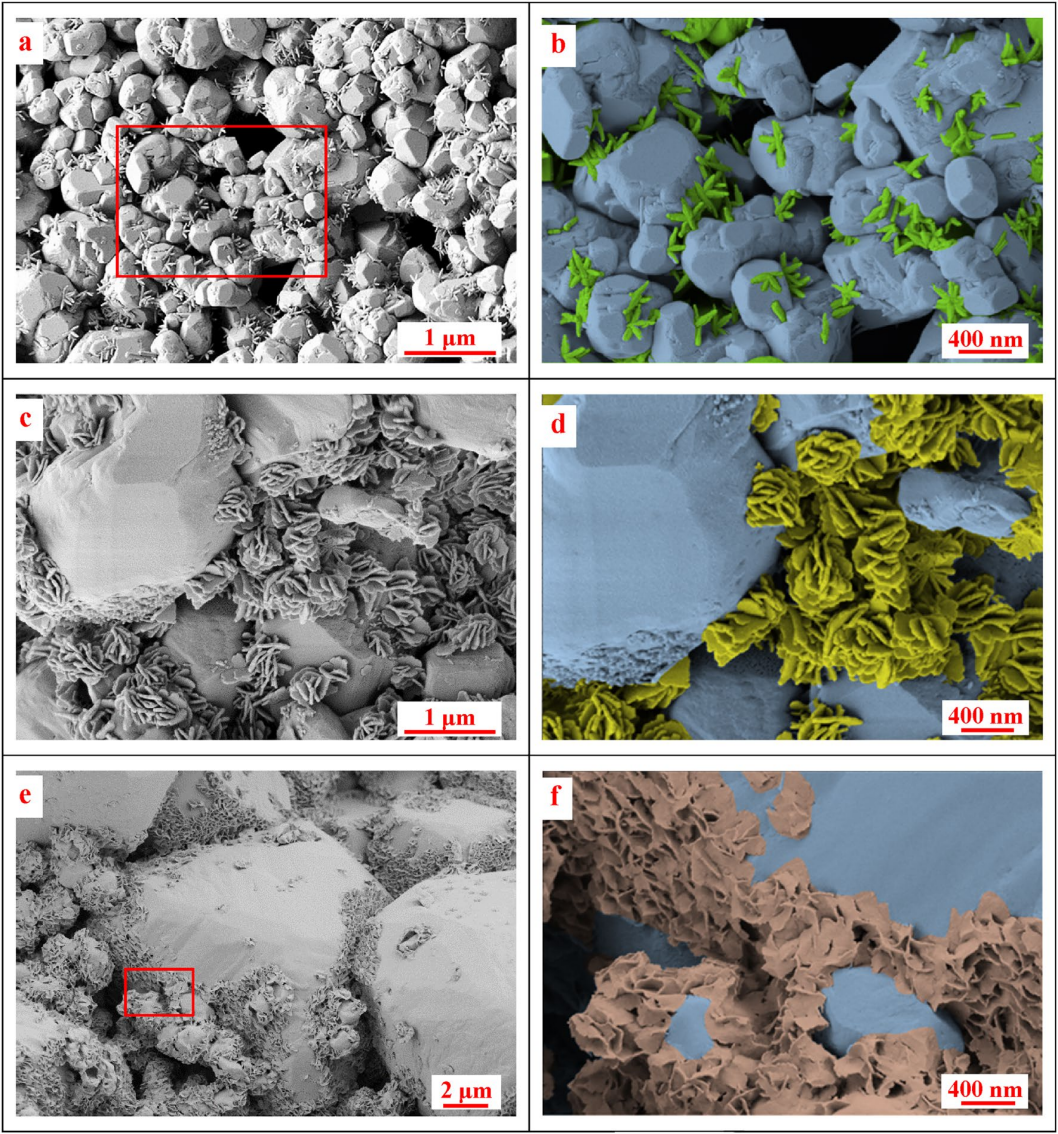
SEM images of (**a**) treated chalk samples at 13.8 MPa and 24 °C showing calcite and needle-like HAP, (**b**) colorized and closer view of image (**a**), (**c**) treated chalk samples at 13.8 MPa and 75 °C, (**d**) colorized and closer view of image (**c**), (**e**) treated chalk samples at 13.8 MPa and 120 °C, and (**f**) colorized and closer view of image (**e**).

Figure 11a,c, and e display uncolored SEM images that reveal the host calcite grains and the distinct HAP crystals of various shapes formed under different conditions. In contrast, Fig. 11b,d, and f show the same SEM images but with pseudo coloring applied to highlight each HAP shape and differentiate them from the host calcite grains. The HAP crystal formations were influenced by variations in the calcite grains' size, shape, and orientation, resulting in different shapes under different pressure and temperature conditions within the same chalk sample. However, certain HAP shapes were more dominant at each pressure and temperature combination. For instance, needle-like, platy hemisphere-like, and sheet-like HAP crystals were observed when chalk reacted with DAP at 13.8 MPa and temperatures of 24, 75, and 120 °C, respectively, as depicted in Fig. 11a,c, and e. The colorized images provide a clear depiction of the formation of needle-shaped HAP (in green), platy hemisphere-shaped HAP (in yellow), and sheet-shaped HAP (in brown), particularly at the contact points between the calcite grains, which appear faded blue, as shown in Fig. 11b,d, and f, respectively. The behavior of the samples treated at 6.9 MPa and various temperatures closely resembled those treated at 13.8 MPa and different temperatures. The assortment of shapes and the most prominent shapes exhibited remarkable similarity under these conditions. The samples subjected to ambient pressure displayed miscellaneous shapes, with sheets being abundant at 24 °C and needles being prominent at 120 °C. However, the crystals formed under these conditions appeared smaller and more sparsely distributed.

## Discussion

The effect of pressure on the elastic stiffness of the chalk samples was not as pronounced as that of temperature, suggesting that temperature plays a more significant role in controlling the chalk's reaction kinetics and resulting mechanical properties. Interestingly, the increase in elastic stiffness at 13.8 MPa was strongly temperature-dependent, with values tripling between 24 and 120 °C. This pronounced enhancement with rising temperature aligns with the literature. As with most chemical reactions, increasing the temperature increases the reaction rate according to the Arrhenius Eq. ^35^. On the contrary, there was no clear trend of the elastic stiffness distribution with increasing the pressure from ambient to 13.8 MPa.

There were two competing factors justifying the small effect of pressure. First, the DAP-calcite reaction yielded carbonic acid (H_2_CO_3_), as emphasized in Eq. 1.

1 $$10{\text{CaCO}}_{3} + 6\left( {{\text{NH}}_{4} } \right)_{2} {\text{HPO}}_{4} + \to ^{{2{\text{H}}_{2} {\text{O}}}} {\text{Ca}}_{10} \left( {{\text{PO}}_{4} } \right)_{6} \left( {{\text{OH}}} \right)_{2} \downarrow + 6\left( {{\text{NH}}_{4} } \right)_{2} {\text{CO}}_{3} + 4{\text{H}}_{2} {\text{CO}}_{{3{ }}}$$

However, carbonic acid is not a stable compound under normal conditions, and it typically decomposes into carbon dioxide (CO_2_) and water (H_2_O) through the following reaction:

2 $${\text{H}}_{2} {\text{CO}}_{{3{ }}} \to ^{ } {\text{CO}}_{2} + {\text{H}}_{2} {\text{O}}$$

Therefore, the partial pressure of carbon dioxide (CO_2_) can affect the equilibrium position of the decomposition reaction^36^. According to Le Chatelier's principle, increasing the partial pressure of CO_2_ shifts the equilibrium of the reaction in the direction that produces more CO_2_. This means that at higher CO_2_ pressures, the equilibrium will favor the left-hand side of the reaction (the solid CaCO_3_) and inhibit the decomposition of calcite. The effect of CO_2_ pressure on the decomposition of calcite is related to the solubility of CO_2_ in water. When CO_2_ is dissolved in water, it forms carbonic acid (H_2_CO_3_), which can react with CaCO_3_ to form soluble calcium bicarbonate (Ca(HCO_3_)_2_). This reaction reduces the concentration of carbonate ions (CO_3_^2−^) in the solution and slows down calcite decomposition. Therefore, at higher CO_2_ pressures, more CO_2_ can dissolve in water, leading to a higher concentration of carbonic acid and a slower decomposition rate. Overall, while pressure does not significantly affect the decomposition of calcite, the partial pressure of CO_2_ can affect the reaction's equilibrium position and the decomposition rate.

Additionally, this study indicates the critical role of reaction duration in determining the mechanical characteristics of chalk. DAP treatment progressively amplified stiffness, reaching over 400% in some samples, with no signs of plateauing, even after 72 h. Interestingly, these observations are consistent with earlier research conducted by Sassoni et al.^37^, who reported comparable reaction kinetics of Indiana limestone treated with DAP solutions at different concentrations. Specifically, the elastic modulus of dry samples attained maturity after 2 days in higher concentrations of 1 and 4.4 M, while it took 4 days in concentrations of 0.1 and 0.5 M at room temperature. Therefore, to achieve optimal mechanical outcomes in chalk, careful consideration of the duration of treatment and DAP concentration is essential. Despite utilizing residual effluents from each cycle in the subsequent cycle, releasing CO_2_ gas substantially impacted the reaction forward, resulting in an increased stiffening effect. This finding further reinforces the ongoing discourse surrounding the influence of CO_2_ partial pressure. Furthermore, this observation serves as compelling evidence, substantiating the superiority of the cycled treatment, conducted for an identical duration of 72 h, in augmenting the stiffening effect on the chalk compared to the continuous treatment.

Furthermore, the conversion of calcium carbonate in the reaction is influenced by factors other than temperature and reaction duration, including the liquid–solid (L/S) ratio, precursor shape, and specific surface area. As per the literature, these parameters play a crucial role in determining the efficiency of the reaction. For instance, Ashokan et al.^18^ found that micron-size calcium carbonate was consumed within 2 h via dissolution precipitation reaction. The process involved adding the calcium carbonate to a solution of DAP, where the Ca/P ratio was kept at 1.67, and the pH was maintained at 10. Comparing porous chalk to calcium carbonate powder, the reaction is expected to be slower due to the limited surface area of the former. A larger surface area allows more reactants to come into contact, resulting in a faster reaction.

Therefore, the rate of the DAP-CaCO_3_ reaction needs to be optimized to achieve the desired outcome for field applications. The catalytic effect observed in chemical reactions is essentially an alternative to temperature's ability to provide additional energy to reactant molecules; as temperature increases, reactant molecules gain more kinetic energy and collide with greater frequency, leading to increased reaction rates. A catalyst provides an alternative route to products with lower energy demand. This phenomenon is well-documented in the academic literature and explains the relationship between temperature and reaction kinetics^38^. As a result, the reactants form the products more quickly. However, the effect of temperature on the DAP-calcite reaction may also depend on other factors, such as the specific conditions of the reaction and the catalytic agents present.

Since porous media have limited reactivity, introducing a catalyst is another way to increase the reaction rate other than temperature. However, it was revealed that LNH has a statistically minor effect on the treated chalk's elastic stiffness.

The minor effectiveness of LNH on the DAP-calcite reaction can be attributed to various factors. One possible reason is the insufficient concentration of the catalyst, which could result in a suboptimal reaction rate. Specifically, the catalyst concentration of 5 g/L may not be adequate to accelerate the reaction effectively. Furthermore, the presence of side reactions may potentially impede the catalyst's effectiveness, particularly when impurities are present.

The XRF findings suggest that HAP was precipitated throughout the sample due to its permeability, allowing the DAP solution to permeate inside. Treatment notably enhanced phosphorus content, increasing average interior intensity from 5 to 8 cps and driving a substantial gradient towards the edges, peaking at 40 cps at the surface.

The concentration gradient can be attributed to the larger volume of solution in direct contact with the edges and the ease of the reaction at the edges compared to the pores' spaces. These results shed light on the behavior of HAP in porous samples and highlighted the importance of considering permeability in such studies. The results agree with the study by Possenti et al.^39^, who utilized Synchrotron radiation µX-ray diffraction to find the depth of treatment. Their results show that the DAP consolidating treatment causes a noteworthy crystallization of Ca and P-bearing compounds several millimeters from the treated limestone surface. Another study by Osticioli et al.^40^ investigated the extent of penetration of ammonium oxalate and DAP on tablets of pure CaCO_3_ and degraded marble samples. The results of the micro-Raman analysis show that a homogeneous distribution of whewellite inside the substrates down to a depth of ~ 1 mm was detected, which became larger in highly degraded regions of the marble substrate. In addition, ca-phosphates in HAP were detected at greater depth (down to 2.5 mm), confirming better consolidating properties of DAP with respect to ammonium oxalate. Anfosso et al.^41^ reported successful treatment in improving the stones' strength up to a depth of 1 mm despite the low DAP concentration. They found that the treated Lecce Stone absorbed less water than untreated ones, and their abrasion resistance was twice as much as the untreated samples up to a depth of 1 mm. However, the abrasion resistance remained constant below a depth of 1 mm, similar to the untreated specimens. Moreover, Franzoni et al.^42^ demonstrated that the DAP treatment, regardless of method (brushing, poultice, immersion), resulted in noticeable enhancements in mechanical properties, particularly in terms of abrasion resistance, indicating its efficacy in strengthening the limestone down to 7.5 mm. These improvements in abrasion resistance are significant as they reflect the stone's cohesion and ability to withstand pulverization^43^.

Calcite-dominant virgin samples transitioned to a surface enriched with 20% HAP following treatment, as evidenced by XRD analysis. The peaks observed for HAP at XRD results are consistent with the characteristic peaks of stoichiometric HAP concerning JCPDS file no. 9-432. The XRD findings are consistent with previous studies in the literature^44^. After analyzing the mineral composition, it was determined that the powder obtained from the near surface of the treated sample contains 20% of HAP. However, it should be noted that the amount of HAP formed varies depending on the location where the powder was extracted from the sample. Also, the XRD finding is consistent with the concentration gradient observed from the XRF analysis. The XRF results indicate that the near-surface has a higher concentration of phosphorous and, consequently, HAP. As a result, the quantification only reflects the relative mineralogy amounts of the extracted piece and not the entire sample.

The different observed HAP shapes were reported in the literature. For instance, Samarkin et al.^24^ conducted a study where they observed the formation of rod-shaped crystals at a temperature of 80 °C. However, in the current study, when pressure was applied, a mixture of needles and rod-like structures was observed at a slightly lower temperature of 75 °C. This finding indicates that pressure might play a role in determining the type of crystals formed. Furthermore, the abundance of minerals formed in this study was lower than in their experiments conducted under normal atmospheric pressure. The observation supports the notion that applying pressure has a negative impact on crystal formation. Additionally, Lin et al.^45^ provided an overview of the latest techniques used to produce calcium phosphate crystals (including HAP) with controlled sizes ranging from nano- to macroscale, as well as varying shapes such as zero-dimensional particles and spheres, one-dimensional rods, fibers, wires, and whiskers, two-dimensional sheets, disks, plates, belts, ribbons, and flakes, and three-dimensional structures.

The reaction conditions, such as temperature, pressure, and reaction duration, can also significantly influence the formation of HAP crystals with different morphologies. For example, a high reaction temperature can lead to the formation of larger HAP crystals. In comparison, a lower temperature can lead to the formation of smaller HAP crystals with different morphologies^34^. On top of that, the micro-heterogeneity of the chalk samples is expected to promote the variation in newly formed minerals. Contrary to the case of using a precursor, It was feasible to control the shape of the HAP crystals generated under hydrothermal conditions by selecting starting materials that raise the level of supersaturation in the solution^46^ or selecting the calcite growth plane^17^. Moreover, Neira et al.^15^ achieved the desired morphology of the HAP crystals by precisely controlling the decomposition rate of urea and the initial component concentration during the hydrothermal process.

In addition, the shape of the crystal can affect its mechanical strength^47^. For example, plate-shaped crystals are generally stronger in compression than needle-shaped crystals, which are stronger in tension. This difference in mechanical strength can affect the overall mechanical properties of the material in which the crystals are embedded, as illustrated in Fig. 3.

## Conclusion

The study explored the use of diammonium phosphate (DAP) as a consolidation agent to improve the strength of weak carbonates for hydraulic fracturing applications. First, the study investigates the effects of pressure, temperature, reaction duration, and catalyst addition on the DAP-calcite reaction and hydroxyapatite (HAP) formation. The experiments showed that temperature and reaction duration are the most critical factors affecting the DAP-calcite reaction, while the pressure effect is minor. Also, the study used XRF to investigate the consolidating phases' crystal chemistry and penetration depth after treating a porous carbonate stone (Austin Chalk) with DAP. According to the investigations, the formation of HAP was consistent throughout the entire sample, and its concentration was found to increase in the last 5 mm towards the surface. The study yields valuable insight into the newly formed HAP at different depths, shedding light on the diffusion mechanism and reactivity of the substrate. The study's findings suggest that DAP is a reliable agent for consolidating weak carbonates and is promising for improving hydraulic fracture conductivity at reservoir conditions. The results can help optimize hydraulic fracturing operations in weak carbonate reservoirs, improving production rates and overall well performance.

## Data Availability

The datasets used and/or analysed during the current study are available from the corresponding author on reasonable request.
